# Arginase Inhibition Ameliorates Hepatic Metabolic Abnormalities in Obese Mice

**DOI:** 10.1371/journal.pone.0103048

**Published:** 2014-07-24

**Authors:** Jiyoung Moon, Hyun Ju Do, Yoonsu Cho, Min-Jeong Shin

**Affiliations:** 1 Department of Food and Nutrition, Korea University, Seoul, Republic of Korea; 2 Department of Public Health Sciences, Graduate School, Korea University, Seoul, Republic of Korea; 3 Korea University Guro Hospital, Korea University, Seoul, Republic of Korea; Nihon University School of Medicine, Japan

## Abstract

**Objectives:**

We examined whether arginase inhibition influences hepatic metabolic pathways and whole body adiposity in diet-induced obesity.

**Methods and Results:**

After obesity induction by a high fat diet (HFD), mice were fed either the HFD or the HFD with an arginase inhibitor, N^ω^-hydroxy-nor-L-arginine (nor-NOHA). Nor-NOHA significantly prevented HFD-induced increases in body, liver, and visceral fat tissue weight, and ameliorated abnormal lipid profiles. Furthermore, nor-NOHA treatment reduced lipid accumulation in oleic acid-induced hepatic steatosis *in vitro*. Arginase inhibition increased hepatic nitric oxide (NO) in HFD-fed mice and HepG2 cells, and reversed the elevated mRNA expression of hepatic genes in lipid metabolism. Expression of phosphorylated 5′ AMPK-activated protein kinase α was increased by arginase inhibition in the mouse livers and HepG2 cells.

**Conclusions:**

Arginase inhibition ameliorated obesity-induced hepatic lipid abnormalities and whole body adiposity, possibly as a result of increased hepatic NO production and subsequent activation of metabolic pathways involved in hepatic triglyceride metabolism and mitochondrial function.

## Introduction

Arginase metabolizes L-arginine to urea and L-ornithine, and has been implicated in the regulation of nitric oxide (NO) synthesis. Arginase potentially inhibits the production of NO by competition with NO synthase (NOS) for the substrate L-arginine [1]. Several lines of evidence have consistently demonstrated that increased arginase activity is associated with endothelial dysfunction in various experimental models of hypertension, atherosclerosis, vascular disease, and aging [2]–[4], and these associations are mainly derived from impaired production of NO. Recently, upregulation of arginase and/or increased arginase activity have also been described in metabolic disorders such as type 2 diabetes mellitus (T2DM) in animals [5], [6] ^,^ and humans [7]. More recently, it was reported that arginase blockade improved endothelial dysfunction in coronary artery disease (CAD) patients with T2DM [8], suggesting arginase as a promising therapeutic target for DM-induced vascular treatment. These studies also reported that the effects were enhanced by increased bioavailability of NO through shunting L-arginine from the arginase pathway to the endothelial NOS pathway [8].

Obesity is a serious health problem that continuously increases the morbidity and mortality of a variety of acute and chronic diseases [9], [10]. Several studies have reported that L-arginine supplementation prevents obesity and obesity-related metabolic complications [11], [12]. Previously, we observed significant upregulations of arginase I in the peripheral blood mononuclear cells (PBMCs) of overweight/obese individuals [13]. In line with this, increased physiological levels of NO could ameliorate all of the adverse features of metabolic complications in obese animals [14]. The apparent metabolic interrelationship between arginine and NO availability raises the question as to whether arginase blockade and the subsequent increase in NO availability might improve obesity and related metabolic conditions.

In the present study, we hypothesized that chronic inhibition of arginase prevents diet-induced obesity. Since previous studies convincingly demonstrated that obesity induces metabolic dysregulation in the liver, such as abnormalities of glucose and lipid homeostasis, which ultimately produces hepatic steatosis [15], we evaluated the effects of the arginase inhibitor N^ω^-hydroxy-nor-L-arginine (nor-NOHA) on hepatic metabolic pathways in obese mice induced by a high fat diet (HFD). This approach ultimately allowed us to test whether arginase inhibition demonstrates an anti-obesity effect. This study provides novel information regarding how arginase inhibition may exert protective properties against hepatic lipid abnormalities induced by obesity and addresses arginase inhibition as a potential therapeutic agent for obesity and related metabolic complications.

## Materials and Methods

### Animals and study design

This animal study was approved by the Institutional Animal Care and Use Committee as governed by the National Institute of Health’s “Guide for the Care and Use of Laboratory Animals and by the Committee on Animal Experimentation and Ethics of Korea University” (KUIACUC-2013-48). Four-week-old male C57BL/6 mice were purchased from DBL (Chungbuk, Korea) and used for the experiment. After a one-week adaptation period, the animals were randomly assigned into control (ND, n = 10), HFD (40% fat for total energy, n = 10), and HFD treated with arginase inhibitor nor-NOHA (HFD with nor-NOHA, n = 10) groups for 12 weeks. Diet composition for each group was presented in Table S1. For obesity induction, all mice in the HFD and HFD with nor-NOHA groups were fed the same HFDs for 7 weeks. These mice were then placed on either only HFD sham gavaged or HFD orally gavaged with 40 mg kg^−1^ nor-NOHA (Bachem; Bubendorf, Switzerland) dissolved in 0.9% NaCl solution for 5 weeks. Daily food intake and weekly body weight gain were routinely recorded throughout the experimental period.

### Sample collection (blood and tissues) and measurement of weight

After 12 weeks, the final body weights were measured and the mice were sacrificed after a 12 h fast. Following this, they were anesthetized with 40 mg kg^−1^ zoletilie (Virbac; Carros, France) mixed with 10 mg kg^−1^ rompun (Bayerkorea; Seoul, Korea); blood samples were collected from the abdominal inferior vena cava, then transferred to vacutainer tubes with EDTA. The centrifuged plasma was aliquoted and stored in a freezer at −80°C until analysis. Liver and visceral adipose tissues (i.e., epididymal, perirenal, retroperitoneal, and mesenteric fat) were extracted, washed with 1X phosphate-buffered saline (PBS), weighed (in g), and then rapidly frozen with liquid nitrogen or put into 10% formaldehyde solution and stored in the freezer at −80°C.

### Analysis of lipid, phosphorylated 5′ adenosine monophosphate-activated protein kinase (p-AMPK)α and histology

Plasma concentrations of total cholesterol (TC), triglyceride (TG), and high-density lipoprotein cholesterol (HDL-c) were measured enzymatically using commercial kits (Asan Pharmaceutical; Seoul, Korea). Hepatic lipids were extracted using the method developed by Folch [16]. The concentrations of TC and TG in the hepatic-lipid extracts were measured using the same enzymatic test kits used for the plasma analysis. p-AMPKα (Thr172) from liver tissue lysates was measured using a commercial kit (Abcam; MA, USA). Tissue samples of the epididymal fat pads and liver were fixed with a 10% formaldehyde solution and embedded in paraffin. Five-millimeter sections were cut, stained with hematoxylin-eosin, viewed with an optical microscope (Nikon; Tokyo, Japan), and photographed at a final magnification of 400×.

### RNA extraction from animal liver and semi-quantitative reverse transcription-polymerase chain reaction (RT-PCR)

To analyze the mRNA expression of genes in liver tissue, total RNA was extracted from 50 mg of liver with the QIAzolLysis reagent of the RNeasy Lipid Tissue Mini Kit (Qiagen; USA) according to the manufacturer’s protocol. cDNA was synthesized from 1 µg of RNA using oligo-dT and Superscript II reverse transcriptase (Invitrogen; USA). One microgram of cDNA was subjected to quantitative real-time PCR amplification using the SYBR Green PCR Kit (Qiagen). The sequences of the designed primers are shown in Table 1. PCR conditions were: 15 min at 95°C, followed by 40 cycles at 94°C for 30 s, 58°C for 20 s, and 7°C for 30 s (Step One Plus; Applied Biosystems; Foster City, CA, USA). GAPDH was used as the control in the comparative cycle threshold (Ct) method.

**Table 1:** Mouse primers used in the experiment.

Gene	Forward primer	Reverse primer
PPAR-γ2	5′-TCATGACCAGGGAGTTCCTC-3′	5′-ACGTGCTCTGTGACGATCTG-3′
SREBP-1c	5′-GGAGGACATCTTGCTGCTTC-3′	5′-CCACAAAGAAACGGTGACCT-3′
ADRP	5′-GTGGAAAGGACCAAGTCTGTG-3′	5′-GACTCCAGCCGTTCATAGTTG-3′
SCD-1	5′-CTTCAAGGGCAGTTCTGAGG-3′	5′-CAATGGTTTTCATGGCAAGTG-3′
ACC-1	5′-GCCTCTTCCTGACAAACGAG-3′	5′-TAAGGACTGTGCCTGGAACC-3′
FAS	5′-CTGAAGAGCCTGGAAGATCG-3′	5′-GTCACACACCTGGGAGAGGT-3′
PPAR-α	5′-GCCACTTGCTCACTACTGTC-3′	5′-AACCAATCAGCTCATGTGAC-3′
CPT-1α	5′-CCAGGCTACAGTGGGACATT-3′	5′-GAAGAGCCGAGTCATGGAAG-3′
PGC-1α	5′-CGGAAATCATATCCAACCAG-3′	5′-TGAGGACCGCTAGCAAGTTTG-3′
PGC-1β	5′-AACCCAACCAGTCTCACAGG-3′	5′-ATGCTGTCCTTGTGGGTAGG-3′
GAPDH	5′-AACTTTGGCATTGTGGAAGG-3′	5′-ACACATTGGGGGTAGGAACA-3′

PPAR, peroxisome proliferator-activated receptor; SREBP-1c, sterol regulatory element-binding protein-1c; ADRP, adipose differentiation-related protein; SCD-1, stearoyl-CoA desaturase-1; ACC-1, acetyl-CoA carboxylase; FAS, fatty acid synthase; PPAR-α, peroxisome proliferator-activated receptor-α; CPT, carnitine palmitoyltransferase; PGC-1, peroxisome proliferator-activated receptor gamma coactivator; GAPDH, Glyceraldehyde 3-phosphate dehydrogenase.

### Cell culture and oleic acid-induced steatosis

The human hepatoma HepG2 cells were obtained from ATCC (American Type Culture Collection, Rockville, MD, USA) and cultured in high-glucose Dulbecco’s modified Eagle medium (DMEM; Gibco; Eggenstein, Germany) supplemented with 10% heat-inactivated fetal bovine serum (FBS) containing 100,000 Units/L penicillamine and 100 mg/L streptomycin (Gibco). The cells were maintained at 37°C in a humidified atmosphere of 95% air and 5% CO_2_. After 24 h, the cells were serum-starved overnight and treated with varying concentrations of oleic acid (OLA; Sigma-Aldrich; St. Louis, MO, USA) for 24 h. Control cells were treated with OLA-free medium containing ethanol.

### Cell viability assay

The HepG2 cells (1×10^5^ cells) were serum-starved overnight and treated with varying concentrations (0–2.0 mM) of OLA (Sigma-Aldrich) for 24 h. Control cells were treated with OLA-free medium containing ethanol. Cell viability was measured by adding 1 mg/mL of 3-(4,5-dmethylthiazol-2-yl)-2,5-diphenyltetrazolium bromide (MTT) to each well and incubating at 37°C for 1 h. After incubation, absorbance was measured at a wavelength of 570 nm with a NanoQuant microplate reader (Tecan Trading AG; Switzerland). This assay was repeated three times.

### Oil Red O staining and quantification of TG content

The HepG2 cells (2×10^5^ cells) were seeded on each well of a 48-well culture plate. After 24 h, the cells were serum-starved overnight. The next day, they were treated with varying amounts of OLA (0, 0.5, 1, and 1.5 mM) or 1.5 mM OLA with nor-NOHA (0, 10, 50 µM) for 24 h. The treated cells were washed with PBS and fixed with 10% formalin for 1 h at room temperature. Subsequently, the cells were washed with 60% isopropanol, stained with Oil Red O for 10 min at room temperature, and washed 4 times with distilled water. Images for each dish were captured using a microscope (Olympus Corporation; Tokyo, Japan). Isopropanol (100%) was added to the cells, and after 10 min, absorbance was measured at 500 nm with a spectrophotometer (PerkinElmer; Waltham, MA, USA). AdipoRed assay reagent (Lonza; Walkersville, MD, USA) was used for the quantification of TG content according to the manufacturer’s protocol.

### Analysis of nitric oxide and western blot

NO from the liver tissue and supernatant of the treated cells was measured using a Nitrate/Nitrite Colorimetric Assay kit (Cayman Chemical; Ann Arbor, MI, USA). Total protein was isolated from liver tissues by homogenization in cold radioimmunoprecipitation assay (RIPA) lysis buffer (Amresco; Solon, OH, USA) containing protease inhibitors (Roche Diagnostics; Mannheim, Germany) and phosphatase inhibitors (Sigma-Aldrich). Total protein was extracted from 1 × 10^6^ HepG2 cells using a cold lysis buffer (40 mM HEPES, pH 7.5, 120 mM NaCl, 1 mM EDTA, 1% Triton X-100) containing a protease inhibitor cocktail (Roche Diagnostics). Western blot analysis was performed using specific antibodies for AMPKα, phospho-AMPKα (Thr172) (Cell Signaling Technology; Danvers, MA, USA) and arginase 1, eNOS, and β-actin (Santacruz Biotechnology, CA, USA).

### Arginase activity assay

Arginase activity was measured using cell lysates and homogenates prepared from liver. The cells and tissues were lysed in cold buffer (50 mM Tris-HCl, PH 7.5, 0.1 mM EDTA, and protease inhibitors) at 4°C and then centrifuged for 20 min at 14,000 g. The supernatants were used using the Quantichrome arginase assay kit (BioAssay Systems, Hayward, CA).

### Statistical analysis

Statistical analysis was performed using SPSS 21.0 (Statistical Package for the Social Science, SPSS Inc.; Chicago, IL, USA). The results are presented as mean ± S.E. and the differences among the experimental groups were analyzed using one-way analysis of variance (ANOVA) with Duncan’s multiple range test and p<0.05 as the criterion of significance.

## Results

### Arginase inhibition prevents diet-induced obesity and reduces liver weight

**Figure 1:**
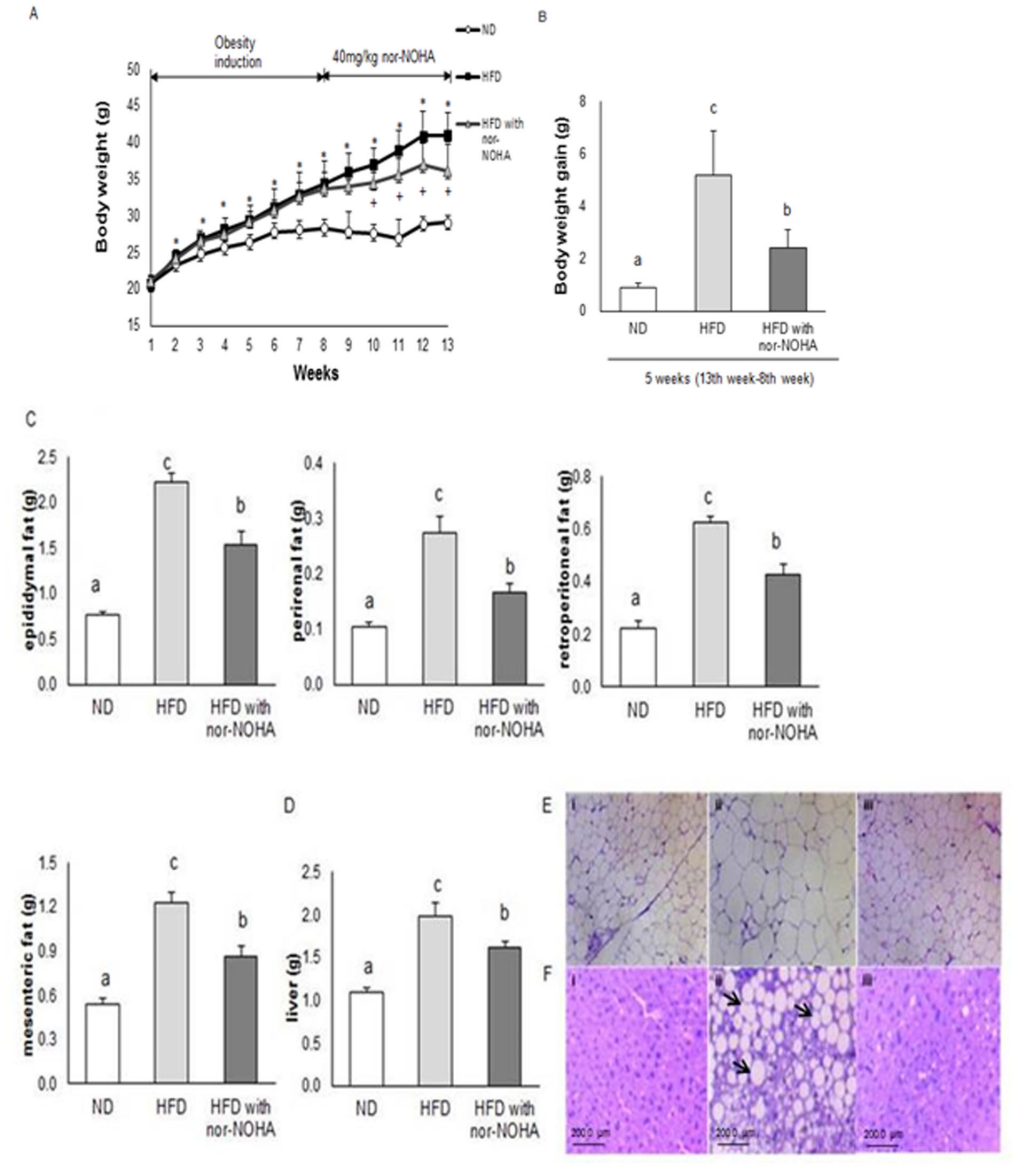
Effects of the nor-NOHA on diet-induced obesity and liver weight. (A) body weight, (B) body weight gain, (C) epididymal, perirenal, retroperitoneal, and mesenteric fat weight, (D) liver weight, (E) epididymal fat morphology, and (F) liver morphology in mice fed with normal or high-fat diets (ND and HFD, respectively). C57BL/6 mice were fed an ND or HFD for 12 weeks. In HFD-fed mice, after 7 weeks of obesity induction, the animals were placed on either only HFD or HFD orally gavaged with 40 mg kg^−1^ nor-NOHA (arginase inhibitor) dissolved in 0.9% NaCl solution for 5 weeks. Body weight was measured weekly. Food intake per mouse was recorded every other day. Representative liver and epididymal fat paraffin sections were stained with hematoxylin and eosin from 3 groups: (i) ND, (ii) HFD, and (iii) HFD with nor-NOHA. Images were captured at 400× magnification. Scale bar, 200 µm. The results are expressed as means ± SEs of mice, and groups were compared by analysis of variance (ANOVA) with Duncan’s multiple range test. The same letter indicates no significant difference between two groups (p<0.05).

As expected, the body weight of HFD-fed mice was significantly increased compared with that of ND-fed mice at the end of the 7-week obesity induction period. There were significant differences in body weight gains between HFD-fed mice and HFD with nor-NOHA-fed mice over the additional 5 weeks (Fig. 1A). HFD with nor-NOHA-fed mice showed a remarkably lower body weight gain compared to mice fed HFD alone (Fig. 1B), although the amount of total food intake was significantly higher in HFD with nor-NOHA-fed mice than in HFD-fed mice. At termination of the 12-week period, the HFD-fed mice showed significantly increased weights in all adipose tissues compared with ND-fed mice (Fig. 1C). HFD with nor-NOHA-fed mice showed significantly reduced adipose tissue weights compared with the HFD-fed mice, and, furthermore, had significantly reduced sizes of epididymal fat adipocytes (Fig. 1E). As for the liver, HFD with nor-NOHA-fed mice showed significantly lower liver weights and marked decreases in lipid accumulation compared to HFD-fed mice (Fig. 1F).

### Effects of arginase inhibition on lipids and NO in plasma and the liver

As shown in Table 2, arginase inhibition by HFD with nor-NOHA significantly decreased the hepatic TG content, whereas it did not affect the hepatic TC content. HFD with nor-NOHA-fed mice displayed significantly lower levels of circulating TG levels compared to those of the control group. With respect to hepatic NO (nitrate+nitrite) concentrations, elevated levels of hepatic NO in HFD-fed mice were even more significantly increased by nor-NOHA (Table 2).

**Table 2:** Effect of nor-NOHA on body weight, food intake, lipid profile and NO in plasma and the liver among the experimental groups.

Groups	ND (n = 10)	HFD (n = 10)	HFD With nor-NOHA (n = 10)
Initial body weight (g)	20.8±1.1	20.6±0.4	21.1±0.5
Final body weight (g)	29.1±1.6^a^	41.0±3.1^c^	36.1±3.6^b^
Food intake (g/day)	2.9±0.02^b^	2.8±0.01^a^	2.9±0.10^b^
FER	0.03±0.01^a^	0.09±0.01^c^	0.06±0.02^b^
Plasma TC (mg/dL)	98.0±6.8	132.9±21.4	99.9±13.2
TG (mg/dL)	83.2±7.4^a^	77.3±7.3^ab^	60.9±4.1^b^
NO (µM)	128.4±12.8	106.0±9.1	118.6±17.9
Hepatic TC (mg/dL)	2.4±0.1^a^	4.8±0.2^b^	4.9±0.2^b^
TG (mg/dL)	12.1±0.6^a^	22.3±1.1^c^	18.2±0.9^b^
NO (µM)	7.1±1.2^a^	16.2±1.4^b^	27.3±3.7^c^

FER: Feed Efficiency Ratio. TC, total cholesterol; TG, triglyceride; NO, nitric oxide. The results were expressed as means ± S.E. of mice tested by analysis of variance (ANOVA) with Duncan’s multiple range test. Sharing the same alphabet (superscript a, b, and c) indicates no significant difference between two groups (p<0.05).

### Effects of arginase inhibition on hepatic lipid metabolism in HFD-fed mice

**Figure 2:**
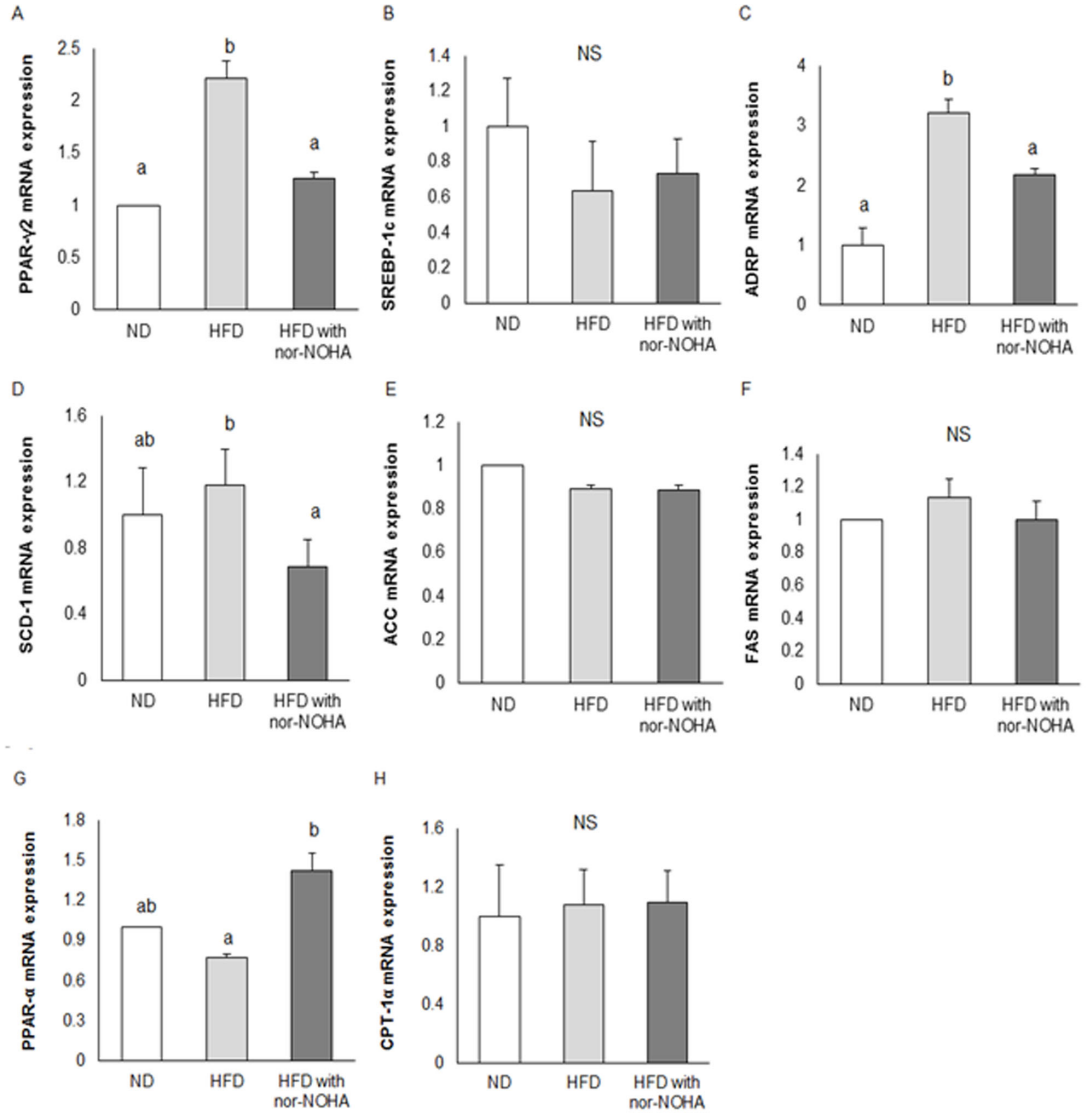
Effects of the nor-NOHA, on the mRNA levels of several genes in mouse livers. Total RNA was extracted from each liver and subjected to real-time PCR analyses using primers specific for PPAR-γ2 (A), SREBP-1c (B), ADRP (C), SCD-1 (D), ACC (E), FAS (F), PPAR-α (G), and CPT-1α (H). The results are expressed as mean ± SE of mice, and differences between groups were tested by analysis of variance (ANOVA) with Duncan’s multiple range test. The same letter indicates no significant difference between two groups (p<0.05).

In order to elucidate the underlying mechanism of metabolic effects of arginase inhibition by nor-NOHA, we first analyzed expression levels of key genes known to control hepatic TG metabolism including *de novo* lipogenesis and fatty acid oxidation. The results showed that the HFD significantly increased mRNA abundance of peroxisome proliferator-activated receptor (PPAR)-γ2 (Fig. 2A), adipose differentiation-related protein (ADRP) (Fig. 2C), and stearoyl-CoA desaturase-1 (SCD-1) (Fig. 2D), and these changes in transcript levels caused by the HFD were reversed by nor-NOHA to levels observed in the ND-fed mice (Fig. 2). By contrast, the HFD did not affect mRNA abundance of sterol regulatory element-binding protein (SREBP) 1_C_ (Fig. 2B), fatty acid synthase (FAS) (Fig. 2F), acetyl-CoA carboxylase (ACC) (Fig. 2E) and carnitine palmitoyltransferase (CPT)-1α (Fig. 2H). In addition, the HFD with nor-NOHA significantly increased hepatic mRNA abundance of PPAR-α compared to the HFD alone (Fig. 2G).

### Effects of arginase inhibition on hepatic mitochondrial function in HFD-fed mice

**Figure 3:**
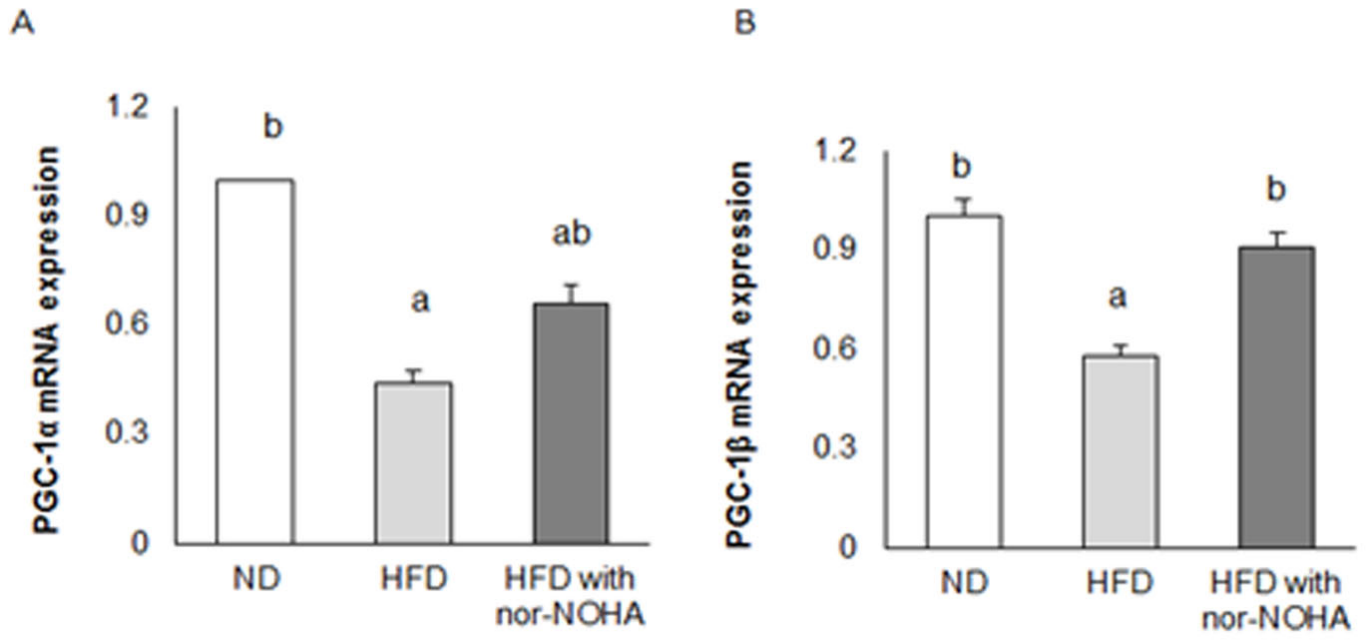
Effects of the nor-NOHA, on the mRNA levels of several genes in the liver. Total RNA was extracted from each liver and subjected to real-time PCR analyses using primers specific for PGC-1α (A), and PGC-1β (B). The results are expressed as mean ± SE of mice, and differences between groups were tested by analysis of variance (ANOVA) with Duncan’s multiple range test. The same letter indicates no significant difference between two groups (p<0.05).

Based on the systemic effects of arginase inhibition on body weight and fat mass, we analyzed the expression of genes involved in hepatic mitochondrial function. The HFD remarkably reduced mRNA abundance of peroxisome proliferator-activated receptor gamma coactivator (PGC)-1α (Fig. 3A) and PGC-1β (Fig. 3B) compared to mice in the ND group, and these changes were reversed by arginase inhibition from nor-NOHA.

### Effects of arginase inhibition on the activation of hepatic AMPKα in HFD-fed mice

**Figure 4:**
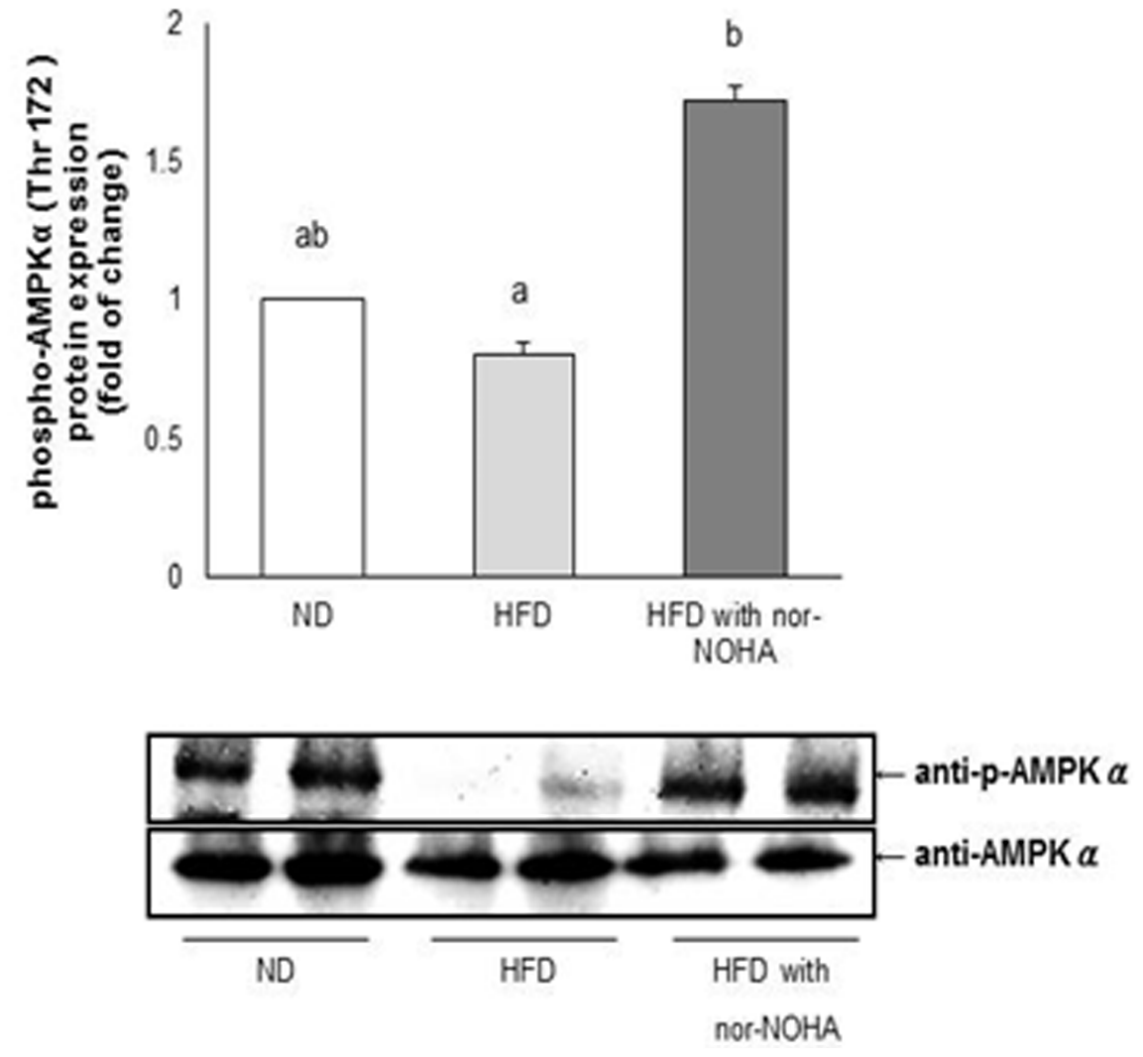
Effect of the nor-NOHA on levels of phosphorylated AMPKα at Thr172 (p-AMPKα) in the liver. Densitometric analysis of p-AMPKα protein levels, using an enzyme-linked immunosorbent assay (ELISA) kit (upper) and protein levels of p-AMPKα and AMPK levels, using a representative western blot image (lower). The results are expressed as mean ± SE of mice, and differences between groups were tested by analysis of variance (ANOVA) with Duncan’s multiple range test. The same letter indicates no significant difference between two groups (p<0.05).

To determine whether the activated AMPKα pathway is required for hepatic mitochondrial function and lipid metabolism improved by arginase inhibition, we investigated that expression of phosphorylated AMPKα at Thr172 in the livers of ND- and HFD-fed mice. Phosphorylation of AMPKα in HFD-fed mice was decreased. However, arginase inhibition dramatically increased phosphorylated AMPKα in HFD with nor-NOHA-fed mice compared to HFD-fed mice (Fig. 4).

### Inhibitory effect of nor-NOHA on lipid accumulation in OLA-induced hepatic steatosis *in vitro*

**Figure 5:**
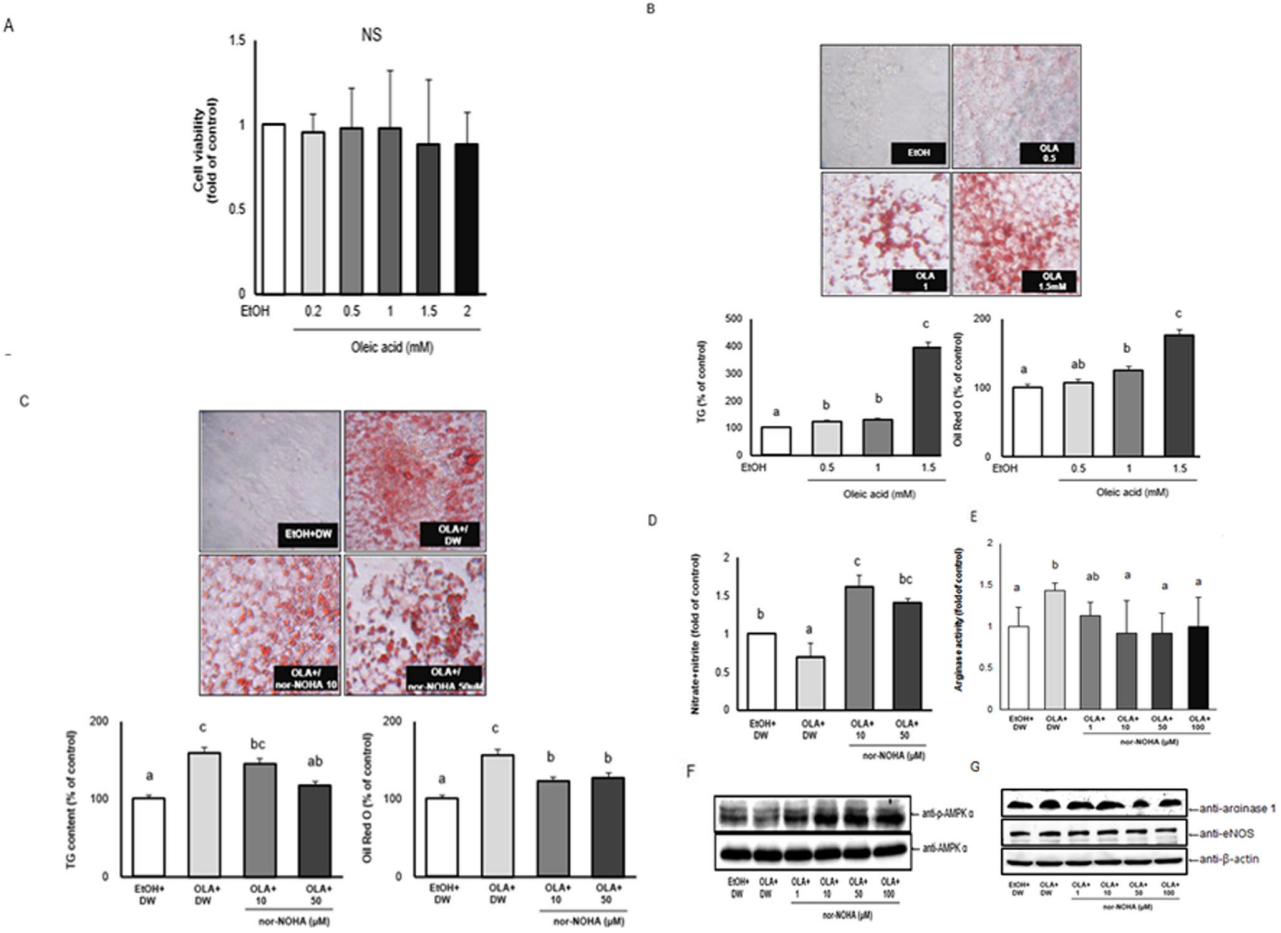
Effect of the nor-NOHA on lipid accumulation in OLA-induced hepatic steatosis in HepG2 cells. (A) Cell viability, (B and C) triglyceride contents, accumulation of intracellular lipid, (D and E) nitric oxide (NO, nitrate+nitrite), arginase activity, and (F and G) protein levels of p-AMPKα, arginase 1, and eNOS. The HepG2 cells were treated with different concentrations of oleic acid (0.2–2.0 mM) or ethanol as a control (CTL) (A and B) or with 1.5 mM oleic acid with nor-NOHA (CTL, distilled water, or 1–100 µM) for 30 min (E) or for 24 h (C, D, F, and G). Cell viability was analyzed using an MTT assay after treatment with different concentrations of oleic acid or ethanol for 24 h. TG content was measured with the AdipoRed assay and intracellular lipids were stained with Oil Red O (400× magnification) and then quantified. The data represent the mean percentage levels compared with ethanol-treated cells. Protein levels of p-AMPKα and AMPK levels, showing a representative western blot. The results are expressed as mean ± SE of at least three independent experimental results, and differences between groups were tested by analysis of variance (ANOVA) with Duncan’s multiple range test. The same letter indicates no significant difference between two groups (p<0.05).

In order to demonstrate that the changes in hepatic phenotypes are not due to secondary weight beneficial effects but are the direct effect of nor-NOHA, we attempted to investigate the direct effects of nor-NOHA in HepG2 cells with OLA-induced hepatic steatosis *in vitro*. To examine the effect of OLA and nor-NOHA on viability in HepG2 cells, first, cells were treated with varying concentrations of OLA and nor-NOHA for 24 h, and then assayed using the MTT assay. The MTT results indicated that treatment of OLA at a dose ranging from 0.2 mM to 2 mM and nor-NOHA at a dose ranging from 1 µM to 100 µM did not affect cell viability (Fig. 5A for OLA, data not shown for nor-NOHA). When the HepG2 cells were cultured at various concentrations of OLA (ethanol, 0.5, 1, 1.5 mM) for 24 h to develop an *in vitro* model of hepatic steatosis, intracellular lipid was accumulated in a dose-dependent manner based on TG content and Oil Red O staining (Fig. 5B). Using a dose of 1.5 mM of OLA, we investigated the effects of nor-NOHA on lipid accumulation, NO release, and AMPKα activation. We found that nor-NOHA treatment markedly diminished the lipid accumulation in HepG2 cells. In addition, nor-NOHA dramatically reduced the intracellular TG content (%) at all doses ranging from 10 µM to 50 µM (Fig. 5C). When the NO level from the supernatant of the treated cells was measured, decreased levels of NO in OLA-induced steatosis HepG2 cells was significantly increased by nor-NOHA at both 10 µM and 50 µM (Fig. 5D). Furthermore, nor-NOHA significantly enhanced phosphorylated AMPKα at a dose of 10 µM (Fig. 5F). Arginase inhibition by nor-NOHA for 30 min reduced arginase activity in OLA- treated HepG2 cells in a dose dependent manner (Fig. 5E), whereas nor-NOHA did not affect the protein expressions of arginase 1 and eNOS in OLA- treated HepG2 cells (Fig. 5G).

## Discussion

Accumulating evidence has demonstrated the role of NO in the regulation of the inter-organ metabolism of energy substrates and the pathological outcomes in metabolic abnormalities [14]. NO, which is synthesized from L-arginine by NOS, participates in multiple cell signaling pathways as a signaling molecule, and therefore regulates the metabolism of energy substrates in a cell- and tissue-dependent manner [1]. Since NOS competes with arginase for L-arginine in producing NO, increased arginase activity and/or arginase-mediated L-arginine depletion provides a mechanism for the metabolic consequences caused by a lack of NO bioavailability. Based on the reciprocal interactions between arginase and eNOS in endothelial dysfunction, increased activity and expression of arginase have been demonstrated in several pathological cardiovascular conditions in T2DM, including hypertension, atherosclerosis, myocardial ischemia, and vascular dysfunction [17]. Although the importance of arginase in endothelial dysfunction and atherosclerosis has been demonstrated, the role of arginase in obesity and obesity-related complications has never been evaluated.

In the present study, we found that nor-NOHA ameliorated hepatic metabolic abnormalities in diet-induced obese mice, and we further demonstrated its systemic effects on body weight and amount of adipose tissue. Specifically, nor-NOHA significantly reduced HFD-induced elevations in hepatic TG content, which was also observed in OLA hepatic steatosis *in vitro*. Moreover, nor-NOHA further increased hepatic NO concentration in HFD-induced obesity, which was also demonstrated in HepG2 cells. In order to elucidate the underlying mechanism for these results, the expression of several genes to control hepatic TG metabolism, including *de novo* lipogenesis and fatty acid oxidation, was investigated.

*SREBP-1c* is the master gene known to be responsible for lipid accumulation in the liver induced by an HFD [18], thus exacerbating hepatic steatosis. On the other hand, several studies have demonstrated that activation of PPAR-γ2 in hepatocytes is sufficient to initiate hepatic steatosis through mechanisms involving activation of lipogenic genes, *de novo* lipogenesis, and increased hepatic TG concentrations [19], even independently of SREBP-1c activation [20]. Although under normal circumstances PPAR-γ2 is only minimally expressed in hepatocytes, it is nonetheless involved in the development of hepatic steatosis by modulating fatty acid transport and the TG incorporation pathway [20]. Enhanced lipogenesis in HFD-fed mice, as shown in the present study, seems to be mainly derived from upregulation of PPAR-γ2, independent of SREBP-1c. This is further supported by the results showing that the mRNAs of target genes for PPAR-γ2, ADRP, and SCD-1 were increased in the livers of HFD-fed mice, whereas SREBP-1c and the mRNAs of its target genes, *FAS* and *ACC*, were not altered. The increased TG accumulation in the livers of HFD-fed mice was likely due to an increase in the expression of *ADRP* and *SCD-1*. Interestingly, nor-NOHA resulted in significant reductions in mRNA levels of PPAR-γ2, ADRP, and SCD-1 in HFD-fed mice without alterations in mRNA levels of SREBP-1c, FAS, and ACC-1, Consistent with previous speculation [21], [22], mRNA abundance of ACC and FAS was not affected in either the HFD- or HFD with nor-NOHA-fed mice, which might be due to a regulatory feedback mechanism in response to HFD feeding. ADRP is the droplet protein where lipids are sequestered in hepatocytes, and its expression is increased in cells expressing PPAR-γ2 [23]. *SCD-1* encodes for the rate-limiting enzyme in monounsaturated fatty acid (MUFA) synthesis, which is a key component in the formation of TG, cholesterol esters, and phospholipids [24]. Our results showing downregulations of ADRP on lipid droplets and SCD-1 by nor-NOHA in the liver indicate that arginase inhibition may exert its inhibitory effects on TG formation and development of fatty liver by modulating ADRP and SCD-1 through regulation of PPAR-γ2. It has been reported that activation of PPAR-α displays antiobesity effects and beneficial effects on the management of hepatic steatosis [25], [26]. It is generally accepted that these effects can be attributed to PPAR-α-induced transcriptional activation of the many genes involved in β-oxidation of fatty acids, which are predominantly found in the liver [27]. Although there was a marked upregulation of PPAR-α in HFD with nor-NOHA-fed mice, mRNA levels of CPT-1α, the oxidative enzyme for fatty acid β-oxidation [28], were not affected. This suggests that reduced hepatic TG accumulation in HFD with nor-NOHA-fed mice probably did not occur through activation of fatty acid β-oxidation.

Defects in mitochondrial energy metabolism have been suggested to cause hepatic steatosis, insulin resistance, and T2DM [29]–[31]. Based on this information, effects of arginase inhibition by nor-NOHA on key genes involved in hepatic mitochondrial bioenergetics were also tested. In the present study, nor-NOHA significantly reversed the reduced PGC-1β expression in HFD-fed mice, whereas the effect on PGC-1α was not significant. PGC-1 is involved in mitochondrial biogenesis, adaptive thermogenesis, fatty acid β-oxidation, and hepatic gluconeogenesis [31], and is a key transcriptional cofactor regulating expression of PPAR-α [29], which in turn stimulates the expression of NRF-1 [31]. In addition, several previous studies have proposed that PGC-1β has a stronger ability to regulate mitochondrial function than PGC-1α [32]. The present study demonstrates that arginase inhibition by nor-NOHA improved mitochondrial function through the upregulation of mainly PGC-1β. It may be that enhanced NO bioavailability resulting from arginase inhibition activates expression of PGC-1 [14], thereby enhancing mitochondrial biogenesis and oxidative phosphorylation. Collectively, it can be speculated that arginase inhibition attenuates HFD-induced hepatic steatosis through modulation in the hepatic expression of genes involved in lipogenesis and mitochondrial function.

Previous studies indicate that AMPKα, which is a member of the metabolic sensor protein kinase family, is involved in regulating vascular function by increasing the activation of eNOS [33] and is downregulated in abnormal metabolic complications such as T2DM and obesity [34]. Indeed, it was reported that vascular eNOS and AMPKα levels are reduced in HFD-fed rats [35]. In addition, AMPKα stimulates expression of PGC-1α [36] and mitochondrial biogenesis [37] as well as fatty acid oxidation [38]. Therefore, it is likely that AMPKα possibly in cooperation with NO [36] plays a key role in regulating lipid metabolism through controlling fatty acid oxidation and activating mitochondrial function. In the present study, the phosphorylation level of AMPKα at Thr172 was significantly up-regulated following arginase inhibition in the livers of HFD-fed mice, suggesting that the NO-AMPKα pathway may be involved in the beneficial effects of arginase inhibition on obesity-induced hepatic abnormalities. This is further supported by our *in vitro* findings clearly showing that arginase inhibition was able to increase NO release and the expression of phosphorylated AMPKα in OLA-induced hepatic steatosis. Also, arginase inhibition by nor-NOHA reduced arginase activity in OLA-treated HepG2 cells in a dose dependent manner, whereas nor-NOHA did not affect the protein expressions of arginase 1 and eNOS. These indicate that the effect of nor-NOHA on NO release was possibly derived from inhibition of arginase activity, not from changes in protein expressions. Of particular interest is the finding that nor-NOHA elicited systemic effects by marked reductions in body weight and fat mass in diet-induced obesity. The mechanism by which arginase inhibition prevents diet-induced obesity was unclear; however, the arginine-NO pathway might be involved in the regulation of whole-body metabolism. Whether the systemic effects shown in the present study were due to arginase inhibition working primarily through enhanced NO production or through an increased bioavailability of arginine remains to be determined, although co-treatment of NOS inhibitor with nor-NOHA has been shown to significantly reverse reduced lipid accumulation by arginase inhibition in OLA-induced hepatic steatosis (Figure S1). Furthermore, since we evaluated the effects of arginase inhibition in established obese animals, we cannot exclude the possibility that longer, chronic arginase inhibition than that tested here could elicit much greater systemic effects.

In conclusion, we found that arginase inhibition showed general improvement in hepatic lipid metabolism and was sufficient to reduce whole body adiposity. The plausible mechanism may be that arginase inhibition increased hepatic NO production, which in turn activated the metabolic pathways involved in hepatic TG metabolism and mitochondrial function. However, caution is needed to interpret data for involvement of NO since we did not test the combined effects of arginase inhibition and NOS inhibition on weight loss. Also, the data regarding the effects of nor-NOHA in a normal diet are required for a clear interpretation of the results. Considering that even a modest weight loss in obese subjects is associated with a significant reduction of cardiovascular risks and diabetes [39], arginase inhibition may be a novel means to reduce body fat, and therefore shows therapeutic potential for human health application.

## Supporting Information

Figure S1 Effect of cotreatment of nor-NOHA and L-NAME on lipid accumulation in OLA-induced hepatic steatosis in HepG2 cells. The HepG2 cells (2×10^5^ cells) were seeded on each well of a 48-well culture plate. After 24 h, the cells were serum-starved overnight. The next day, they were treated with 1.5 mM OLA co-treated with 5 µM nor-NOHA using 25 µM L-NAME (N G-nitro-L-arginine methyl ester) for 24 h. The co-treated cells were washed with PBS and fixed with 10% formalin for 1 h at room temperature. Subsequently, the cells were washed with 60% isopropanol, stained with (B) Oil Red O for 10 min at room temperature, and washed 4 times with distilled water. Images for each dish were captured using a microscope (400× magnification, Olympus Corporation; Tokyo, Japan). Isopropanol (100%) was added to the cells, and after 10 min, absorbance was measured at 500 nm with a spectrophotometer (PerkinElmer; Waltham, MA, USA). (A) AdipoRed assay reagent (Lonza; Walkersville, MD, USA) was used for the quantification of TG content according to the manufacturer’s protocol. The data represent the mean percentage levels compared with ethanol-treated cells. The results are expressed as mean ± SE of at least three independent experimental results, and differences between groups were tested by analysis of variance (ANOVA) with Duncan’s multiple range test. The same letter indicates no significant difference between two groups (p<0.05). The result showed that co-treatment of 25 µM L-NAME with nor-NOHA significantly reversed reduced lipid accumulation in OLA-induced hepatic steatosis by arginase inhibition.
(DOCX)

Table S1 Composition of experimental diets.
(DOCX)
