# Supplementary material for: Arginase Inhibition Ameliorates Hepatic Metabolic Abnormalities in Obese Mice
Source: PLoS One. 2014 Jul 24;9(7):e103048. doi: 10.1371/journal.pone.0103048 (PMC4109998; doi:10.1371/journal.pone.0103048)
Supplement: Figure S1 — Effect of cotreatment of nor-NOHA and L-NAME on lipid accumulation in OLA-induced hepatic steatosis in HepG2 cells. The HepG2 cells (2×10^5^ cells) were seeded on each well of a 48-well culture plate. After 24 h, the cells were serum-starved overnight. The next day, they were treated with 1.5 mM OLA co-treated with 5 µM nor-NOHA using 25 µM L-NAME (N G-nitro-L-arginine methyl ester) for 24 h. The co-treated cells were washed with PBS and fixed with 10% formalin for 1 h at room temperature. Subsequently, the cells were washed with 60% isopropanol, stained with (B) Oil Red O for 10 min at room temperature, and washed 4 times with distilled water. Images for each dish were captured using a microscope (400× magnification, Olympus Corporation; Tokyo, Japan). Isopropanol (100%) was added to the cells, and after 10 min, absorbance was measured at 500 nm with a spectrophotometer (PerkinElmer; Waltham, MA, USA). (A) AdipoRed assay reagent (Lonza; Walkersville, MD, USA) was used for the quantification of TG content according to the manufacturer’s protocol. The data represent the mean percentage levels compared with ethanol-treated cells. The results are expressed as mean ± SE of at least three independent experimental results, and differences between groups were tested by analysis of variance (ANOVA) with Duncan’s multiple range test. The same letter indicates no significant difference between two groups (p<0.05). The result showed that co-treatment of 25 µM L-NAME with nor-NOHA significantly reversed reduced lipid accumulation in OLA-induced hepatic steatosis by arginase inhibition. (DOCX) [file pone.0103048.s001.docx]

Figure S1.

B

A
