# Supplementary material for: Arginase Inhibition Ameliorates Hepatic Metabolic Abnormalities in Obese Mice
Source: PLoS One. 2014 Jul 24;9(7):e103048. doi: 10.1371/journal.pone.0103048 (PMC4109998; doi:10.1371/journal.pone.0103048)
Supplement: Table S1 — Composition of experimental diets. (DOCX) [file pone.0103048.s002.docx]

Table S1. Composition of experimental diets

(g/100g diet)

| Ingredients | ND | HFD | HFD  with nor-NOHA |
| --- | --- | --- | --- |
| corn starch | 15 | 15 | 15 |
| casein | 20 | 20 | 20 |
| sucrose | 50 | 34 | 34 |
| corn oil | 5 | 3 | 3 |
| Mineral mix^1^ | 3.5 | 3.5 | 3.5 |
| Vitamin mix^2^ | 1 | 1 | 1 |
| Cellulose | 5 | 5 | 5 |
| DL-methionine | 0.3 | 0.3 | 0.3 |
| choline bitartrate | 0.2 | 0.2 | 0.2 |
| Lard | - | 17 | 17 |
| cholesterol | - | 1 | 1 |
| BHT | 0.001 | 0.001 | 0.001 |
| total | 100 | 100 | 100 |

^1^AIN-76 mineral mix. ^2^AIN-76 vitamin mix. BHT, butylated hydroxytoluene; nor-NOHA, Nω-hydroxy-nor-Arginine; ND, normal diet; HFD, high fat diet.
